# Increased risk of peripheral arterial disease in polymyalgia rheumatica: a population-based cohort study

**DOI:** 10.1186/ar2664

**Published:** 2009-03-31

**Authors:** Kenneth J Warrington, Elena P Jarpa, Cynthia S Crowson, Leslie T Cooper, Gene G Hunder, Eric L Matteson, Sherine E Gabriel

**Affiliations:** 1 Division of Rheumatology, Department of Medicine, Mayo Clinic, 200 First Street SW, Rochester, MN 55905, USA; 2 Division of Biostatistics, Department of Health Sciences Research, Mayo Clinic, 200 First Street SW, Rochester, MN 55905, USA; 3 Division of Cardiovascular Diseases, Department of Medicine, Mayo Clinic, 200 First Street SW, Rochester, MN 55905, USA; 4 Division of Epidemiology, Department of Health Sciences Research, Mayo Clinic, 200 First Street SW, Rochester, MN 55905, USA

## Abstract

**Introduction:**

The present study was conducted to determine whether patients with polymyalgia rheumatica (PMR) are at an increased risk of peripheral arterial disease (PAD).

**Methods:**

An inception cohort of all Olmsted County, Minnesota residents diagnosed with PMR between 1 January 1970 and 31 December 1999 was compared with non-PMR subjects (two for each PMR subject) from among residents. Both cohorts were followed longitudinally by complete medical record review from the incidence date of PMR (or index date for the non-PMR cohort) until death, incident PAD, migration, or 31 December 2006. PMR-related disease characteristics, traditional cardiovascular risk factors and diagnosis of PAD were abstracted from the medical record. Cumulative incidence of PAD was estimated using Kaplan–Meier methods. Cox proportional hazards models were used to assess the risk of PAD in PMR compared with non-PMR.

**Results:**

A total of 353 PMR patients (mean age 73.3 years, 67% women) and 705 non-PMR subjects (mean age 73.2 years, 68% female) were followed for a median of 11.0 years. PAD developed in 38 patients (10-year cumulative incidence, 8.5%) with PMR and in 28 non-PMR subjects (10-year cumulative incidence, 4.1%) (hazard ratio (95% confidence interval), 2.40 (1.47, 3.92)). After adjusting for traditional cardiovascular risk factors, patients with PMR still had a significantly higher risk for PAD (hazard ratio, 2.50 (1.53, 4.08)) compared with controls. Giant cell arteritis occurred in 63 (18%) PMR patients but was not predictive of PAD (*P *= 0.15). There was no difference between mortality in PMR and the non-PMR cohorts nor in PMR patients with and those without PAD (*P *= 0.16).

**Conclusions:**

Patients with PMR appear to have an increased risk of PAD.

## Introduction

Polymyalgia rheumatica (PMR) is an inflammatory condition affecting middle-aged and older persons that is characterized by aching and stiffness, typically in the cervical region, shoulders, hips and proximal extremities. Most patients with PMR have laboratory evidence of an acute phase response, including elevation of the erythrocyte sedimentation rate and C-reactive protein (CRP). Synovitis in proximal joints and periarticular structures appears to be responsible for the musculoskeletal symptoms in this condition [1-3]. Approximately 16 to 21% of patients with PMR develop giant cell arteritis (GCA), an inflammatory vasculopathy that affects large and medium-size arteries. The vascular bed of the extracranial arteries is typically involved in GCA, but the aorta and its primary and secondary branches can also be affected [3,4]. PMR is characteristically very responsive to treatment with corticosteroids; however, some patients have a chronic, relapsing course that lasts for several years [5].

Some chronic inflammatory disorders such as rheumatoid arthritis and systemic lupus erythematosus are associated with an increased incidence of cardiovascular disease, thought to be due to the effect of inflammation on progression of atherosclerosis [6-8]. Similarly, there are data to suggest that PMR is also associated with an increased risk of cardiovascular disease [9].

Clinically evident peripheral arterial disease (PAD) is common among older people and the prevalence of this condition increases markedly with age, ranging from 2.5% for those between the ages of 50 and 59 up to 14.5% in those aged 70 and older [10]. Among the traditional cardiovascular risk factors, diabetes mellitus and tobacco use are the strongest risk factors for the development and progression of PAD. Hypertension and dyslipidemia are weaker risk factors [11]. Inflammation is also a recognized risk factor for PAD. In particular, elevated fibrinogen and CRP levels have also been associated with PAD [10].

We hypothesized that chronic inflammation due to PMR may be associated with an increased risk of PAD. The present study was undertaken to examine the incidence of clinically significant PAD in patients with PMR compared with a control cohort from the same geographic area. Risk factors for PAD and the effect of PAD on survival were also evaluated.

## Materials and methods

The population-based retrospective cohort study among residents of Olmsted County, Minnesota was approved by the Mayo Clinic and Olmsted Medical Center Institutional Review Boards. Since this is a retrospective study that poses minimal risk to subjects, the Institutional Review Boards granted a waiver of written informed consent for this study. All study subjects, however, had authorized access to their medical record for research purposes.

### Polymyalgia rheumatica cohort

An inception cohort of all Olmsted County residents first diagnosed with PMR between 1 January 1970 and 31 December 1999 was assembled. This cohort has been described previously [5,9,12]. Individuals were included as subjects with PMR if they fulfilled the following three criteria: age ≥ 50 years; bilateral aching and morning stiffness (lasting 30 minutes or more) persisting for at least 1 month involving two areas from the neck or torso, the shoulders or proximal regions of the arms, and the hips or proximal aspects of the thighs; and an erythrocyte sedimentation rate >40 mm/hour (erythrocyte sedimentation rate, Westergren method). Patients with suggestive clinical findings who fulfilled the first two of the three criteria, and who had a prompt response (definite improvement in symptoms within 24 hours) to low-dose corticosteroid therapy (20 mg prednisone/day or less), were also considered to have PMR. The presence of another disease that could explain the symptoms, such as active rheumatoid arthritis, was considered an exclusion criterion. A concomitant diagnosis of GCA was documented if subjects fulfilled the 1990 American College of Rheumatology criteria [13].

### Non-polymyalgia rheumatica cohort

A comparison cohort of non-PMR subjects (two subjects for each PMR subject) was also assembled from among Olmsted County residents. Each non-PMR subject had a similar birth year (± 3 years), sex, and length of medical history as the PMR subject. Each subject in the non-PMR cohort was assigned an index date corresponding to the PMR incidence date (baseline) of the corresponding PMR subject.

Both the PMR and non-PMR cohorts were followed longitudinally by complete (inpatient and outpatient) medical record review, starting at the incidence date of PMR (or the index date for the non-PMR cohort) and continuing until death, incident PAD, migration, or 31 December 2006. PMR-related disease characteristics were abstracted from the medical record, including the following variables: aching neck, aching shoulders/arms, aching hip/thigh, pain distal to arms, pain to the chest/abdomen, pain to upper/lower back, tenderness in upper arms/shoulders, tenderness in hips/thighs, tenderness in elbow/wrist/hand/finger, tenderness in knee/leg/foot/ankle/toe, anorexia, malaise/fatigue, weight loss. The diagnosis of PAD was abstracted from the medical record. PAD was defined as present if one of the following criteria were met: ankle-brachial index ≤ 0.90 in either leg; symptoms of intermittent claudication or ischemic pain at rest with examination findings including documented absence of pedal and posterior tibial artery pulses or bruits over the femoral arteries; presence of prior peripheral artery surgery or lower extremity amputation for PAD; aortoiliac stenosis on imaging studies (magnetic resonance angiography, computed tomography angiography, conventional angiography).

An ankle-brachial index ≤ 0.90 is 90% sensitive and 95% specific for PAD [14]. We used a clinical definition of PAD to avoid missing cases that may not have undergone noninvasive arterial studies. The above criteria for PAD have been used individually or in combination in previous epidemiological studies of PAD [15].

Diagnoses of traditional cardiovascular risk factors (diabetes mellitus, hypertension and dyslipidemia) were obtained from diagnostic indices using the ICD9 codes (diabetes, 250; hypertension, 401 to 405; dyslipidemia, 272).

### Statistical analysis

Descriptive statistics (means, standard deviations, proportions) were used to summarize the data. The cumulative incidence of PAD and survival following PAD were estimated using Kaplan–Meier methods [16]. Cox proportional hazards models were used to assess the risk of PAD in PMR subjects compared with non-PMR subjects, as well as to examine the influence of PMR disease characteristics on the development of PAD within the PMR cohort. A time-dependent covariate was used to examine the effect of GCA in order to account for development of GCA during follow-up. Similarly, time-dependent covariates were used to adjust for diabetes mellitus, hypertension and dyslipidemia.

## Results

The PMR cohort included 364 patients while the non-PMR cohort consisted of 728 individuals (two per case). We excluded any individuals (from either cohort) with a diagnosis of PAD prior to study entry. Our study population therefore included a total of 353 PMR patients (mean age 73.3 years, 67% women) and 705 non-PMR subjects (mean age 73.2 years, 68% women). There was no significant difference at baseline in the prevalence of diabetes mellitus, hypertension and dyslipidemia between the PMR and the non-PMR cohorts (Table 1).

**Table 1:** Demographics and risk factors in polymyalgia rheumatica (PMR) patients and non-PMR patients

Variable	Non-PMR (n = 705)	PMR (n = 353)	*P *value
Age (years)	73.2 ± 8.6	73.3 ± 8.6	
Follow-up (years)	10.2 ± 7.4	11.7 ± 6.5	
Female	476 (68%)	237 (67%)	
Diabetes mellitus	170 (24%)	91 (26%)	0.55
Hypertension	470 (67%)	248 (70%)	0.24
Dyslipidemia	228 (32%)	127 (36%)	0.24

Data presented as mean ± standard deviation or *n *(%).

**Figure 1:**
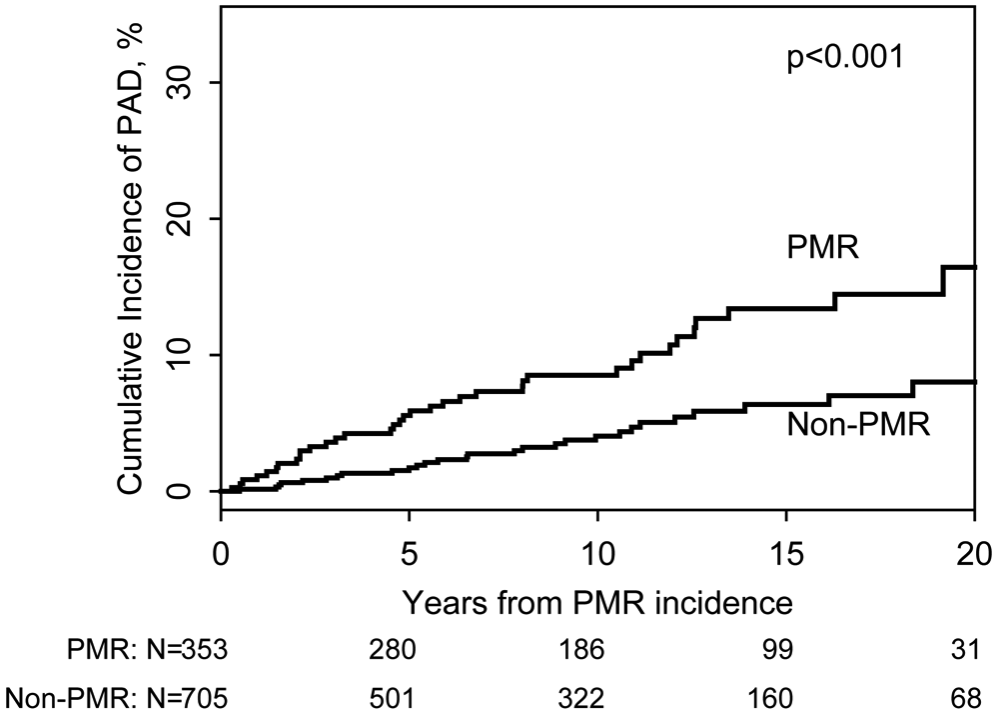
Cumulative incidence of peripheral arterial disease in the study cohorts. Cumulative incidence of peripheral arterial disease (PAD) in 353 polymyalgia rheumatica (PMR) patients and 705 non-PMR patients.

These individuals were followed for a median of 11.0 years. During the follow-up period, 38 patients (10-year cumulative incidence, 8.5%) with PMR developed PAD while 28 of the non-PMR subjects (10-year cumulative incidence, 4.1%) developed PAD (hazard ratio (95% confidence interval), 2.40 (1.47, 3.92)) (Figure 1).

After adjusting for hypertension, dyslipidemia, and diabetes mellitus, patients with PMR still had a significantly higher risk for PAD (hazard ratio (95% confidence interval), 2.50 (1.53, 4.08)) compared with those who did not have PMR (Table 2).

**Table 2:** Risk of developing peripheral arterial disease in polymyalgia rheumatica and non-polymyalgia rheumatica

Model adjustors	Hazard ratio (95% confidence interval)
Age and sex	2.40 (1.47, 3.92)
Age, sex, diabetes mellitus, hypertension, dyslipidemia	2.50 (1.53, 4.08)

All patients with PAD (both in the PMR group and the non-PMR group) had symptoms of lower extremity claudication, and the majority had abnormal pedal pulses. The use of non-invasive Doppler vascular studies and the prevalence of abnormal ankle-brachial indexes were similar in both groups of patients. PMR patients with PAD were less likely to undergo vascular imaging studies (*P *= 0.01). It is therefore unlikely that our results are due to detection bias related to increased surveillance for PAD in the PMR cohort. Owing to the small sample size of patients undergoing detailed imaging studies, we were unable to determine whether there is a difference in severity of PAD in PMR patients versus non-PMR patients. PMR patients with PAD, however, underwent less revascularization procedures compared with the non-PMR cohort (*P *= 0.038) (Table 3).

**Table 3:** Characteristics and imaging studies of peripheral arterial disease in polymyalgia rheumatica (PMR) and non-PMR cohorts

Variable	Non-PMR cohort (n = 28)	PMR cohort (n = 38)	*P *value
Claudication symptoms	28 (100%)	38 (100%)	
Dorsalis pedis pulse			0.09
Abnormal	24 (92%)	37 (100%)	
Normal	2 (8%)	0 (0%)	
Noninvasive arterial study done	17 (61%)	19 (50%)	0.39
Ankle-brachial index < 0.90 in either leg	13 (76%)	17 (89%)	0.30
Computed tomography angiogram done	12 (43%)	5 (13%)	0.006
Magnetic resonance angiogram done	8 (29%)	3 (8%)	0.026
Conventional angiogram done	11 (39%)	6 (16%)	0.031
Any vascular imaging done	14 (50%)	8 (21%)	0.014
Stenosis on imaging			0.08
Yes	5 (36%)	6 (75%)	
No	9 (64%)	2 (25%)	
Revascularization for peripheral arterial disease	9 (32%)	4 (11%)	0.038
Percutaneous intervention/stent	2 (7%)	0 (0%)	0.09
Vascular bypass surgery	6 (22%)	4 (11%)	0.21
Amputation for peripheral arterial disease	2 (7%)	1 (3%)	0.40

Data presented as *n *(%).

We did not identify any PMR-related disease characteristics (for example, location of musculoskeletal symptoms, constitutional symptoms) that were significantly associated with the development of PAD (data not shown).

Since inflammation may be playing a role in progression of atherosclerosis, we examined whether the erythrocyte sedimentation rate (as a continuous variable) at the time of PMR diagnosis was associated with development of PAD. The erythrocyte sedimentation rate value closest to the PMR diagnosis date (data for approximately 98% of patients was available) was not predictive of PAD development.

While receiver operating characteristic analyses could shed further light on this relationship, the data were inadequate to support such an analysis. Concomitant GCA was not significantly associated with the development of PAD in this cohort (*P *= 0.15). A total of 63 (18%) PMR patients had GCA.

**Figure 2:**
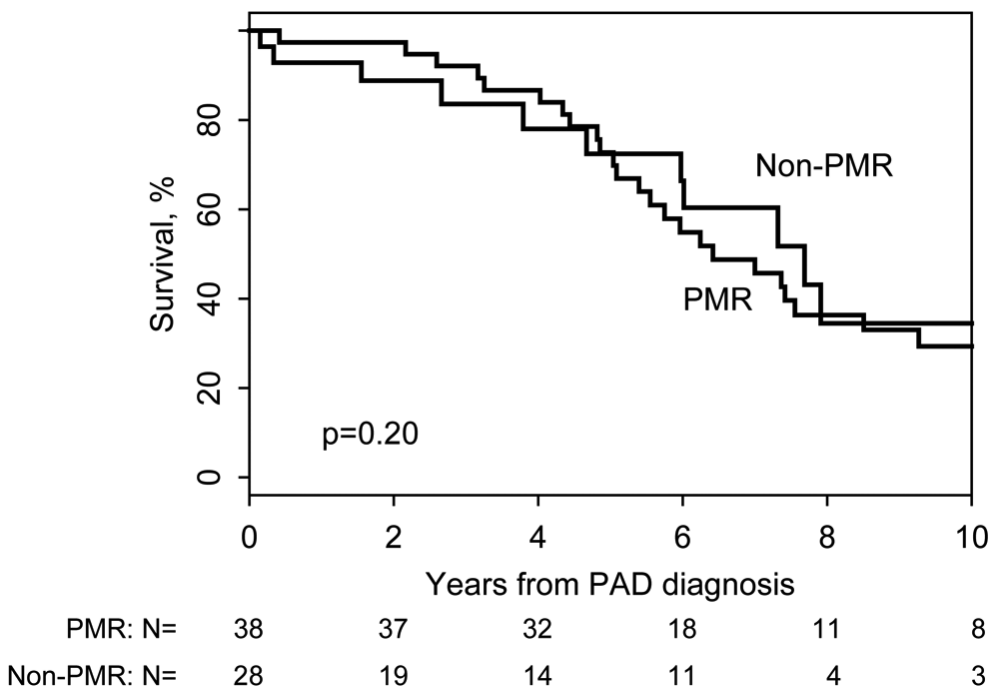
Survival following development of peripheral arterial disease in the study cohorts. Survival following development of peripheral arterial disease (PAD) in 38 polymyalgia rheumatica (PMR) patients and 28 non-PMR patients.

There was no difference between overall mortality in PMR patients compared with the non-PMR cohort. In addition, and as previously reported, there was no difference in survival for PMR patients comparing those with PAD with those without PAD (*P *= 0.16). Similarly, among those individuals who developed PAD, a prior diagnosis of PMR had no effect on survival (*P *= 0.20) (Figure 2).

## Discussion

Results from this population-based cohort study suggest that individuals with PMR have an increased risk of PAD. This increased risk may be due to premature atherosclerosis related to chronic inflammation. Alternatively, PAD in patients with PMR may represent unrecognized or subclinical vasculitis.

Inflammation clearly plays a major role in the pathogenesis of atherosclerosis. Indeed, chronic inflammatory conditions such as rheumatoid arthritis and systemic lupus erythematosus are associated with accelerated atherosclerosis and premature coronary artery disease [6-8]. Chronic inflammation is considered a major risk factor for both coronary and noncardiac vascular disease in rheumatoid arthritis [17].

Among apparently healthy men, baseline levels of CRP predict future risk of developing symptomatic PAD [18]. In a recent, prospective study, however, CRP at baseline in patients with established PAD was not predictive of disease progression over a 3-year follow-up period [19]. Another study concluded that high-sensitivity CRP was predictive of large-vessel PAD [20]. Higher CRP levels are associated with greater functional impairment in patients with PAD [21]. In the general population, patients who have PAD and increased inflammation are at high risk for adverse cardiovascular outcomes [22].

Overt vasculitis occurs in PMR and GCA. In these cases, direct vascular immune-mediated injury may account for the increased incidence of PAD in this patient population. While we did not detect an association between concomitant GCA and the risk of PAD, we cannot definitely rule out such an association due to limited statistical power. It is possible that the incidence of concomitant GCA, which rarely clinically involves the lower extremity arteries, was underestimated in this cohort. Biopsy-proven GCA can occasionally occur in patients without cranial symptoms [23].

PMR patients without clinically overt GCA may also frequently have subclinical vasculitis, and therefore the complications of GCA may also be encountered in patients with PMR. Indeed, Weyand and colleagues demonstrated the presence of macrophage-derived and T-cell-derived cytokines in temporal artery biopsy specimens from patients with PMR but without arteritis [24]. Activation and cytokine production from circulating monocytes is common to both patients with GCA and PMR [25].

Using fluorodeoxyglucose positron emission tomography scanning, Blockmans and colleagues found that three out of five patients with PMR had increased fluorodeoxyglucose uptake in the lower extremity vasculature, suggesting that subclinical or asymptomatic vasculitis is frequently present in PMR [26]. This was confirmed in a similar, more recent, study [27].

The strength of the present study is that it takes advantage of the population-based medical data available through the Rochester Epidemiology Project resources [28,29]. This linked medical-records system allows access to accurate and detailed clinical and laboratory data over many years, which are not typically available in other databases or research settings [30]. The primary limitation of the present study is due to its retrospective nature. This study relies on complete and accurate recording of pertinent information in the medical record. Several major cardiovascular risk factors have been incorporated into our analysis. Other elements, such as complete medication (steroid) usage and smoking history, were not analyzed. In a recent study, however, use of glucocorticoids for PMR was not associated with increased cardiovascular events and is unlikely to account for our findings [31]. Specifically, Maradit-Kremers and colleagues reported that glucocorticoid exposure in patients with PMR was not associated with a higher risk of PAD. Moreover, timing of glucocorticoid exposure and cumulative glucocorticoid dose was also not associated with a greater risk of PAD in these patients [31].

It should be noted that our findings are applicable primarily to the US white population since the Olmsted County population during the time period of the study was >95% white.

A significant number of patients with PAD are asymptomatic and therefore we may have underestimated the incidence of PAD in our study. This potential detection bias should be equal for the PMR cohort and the non-PMR cohort, however, particularly since it has not been previously suspected that patients with PMR are at higher risk for PAD and physicians would therefore not have made more effort to detect PAD in this cohort. Moreover, our focus was on clinically significant, symptomatic PAD that could impact the patients' quality of life – as opposed to asymptomatic disease.

The present study adds to the growing body of literature pertaining to the risk of cardiovascular disease in chronic inflammatory conditions. Patients with PMR and PAD should be monitored closely and modifiable cardiovascular risk factors should be managed aggressively.

## Conclusions

Patients with PMR appear to have an increased risk of PAD. Future studies will be required to understand the mechanisms underlying the excess risk of PAD in PMR in order to identify and optimally treat at-risk PMR patients.

## Abbreviations

CRP: C-reactive protein; GCA: giant cell arteritis; PAD: peripheral arterial disease; PMR: polymyalgia rheumatica.

## Competing interests

The authors declare that they have no competing interests.

## Authors' contributions

KJW participated in the design of the study, reviewed medical records, and drafted the manuscript. EPJ reviewed medical records for acquisition of data and was involved in drafting the manuscript. CSC participated in the design of the study and performed the statistical analyses. LTC participated in the design of the study and revised the manuscript for important intellectual content. GGH participated in the design of the study and revised the manuscript for important intellectual content. ELM participated in the design of the study and data interpretation, and revised the manuscript for important intellectual content. SEG conceived of the study, and participated in its design and coordination and helped to draft the manuscript. All authors read and approved the final manuscript.
